# 2547: Sinai MedMaker Challenge: A model of experiential team science education

**DOI:** 10.1017/cts.2017.186

**Published:** 2018-05-10

**Authors:** Peter Backeris, Janice Lynn Gabrilove, Caroline Eden, Crispin Goytia, Kevin Costa

**Affiliations:** Icahn School of Medicine at Mount Sinai, New York, NY, USA

## Abstract

OBJECTIVES/SPECIFIC AIMS: Innovation in healthcare is increasingly dependent on technology and teamwork, requiring effective collaboration among diverse disciplines. However, large knowledge barriers exist between these diverse disciplines which hinders effective communication and the innovation processes. We organized an intensive team-based competition event, Sinai MedMaker Challenge, that engaged individuals with a wide range of backgrounds in medicine, biomedical research, computers science, and engineering to collaborate in solving medical problems with technology-based solutions. The learning objectives were to: enable participants to identify healthcare problems which lend themselves to technology-based solutions; delineate key behaviors critical to multidisciplinary team success; identify optimal strategies for communicating in teams; engage and inspire participants to apply knowledge of technology to meaningfully impact clinical care and well-being. METHODS/STUDY POPULATION: The Sinai MedMaker Challenge was a 48-hour team-based competition, modeled after previously held health “hackathons.” Adapting from guidelines provided by MIT Hacking Medicine, the event gathered participants from diverse backgrounds (clinicians, medical students, graduate students in biomedical science and humanities, software developers, engineers, and others), for the purpose of utilizing technology to address pressing problems in the diagnosis, management and/or treatment of pain and/or fatigue. The event flow can be outlined as follows: Phase 1—pre-event brainstorming via Slack and Sparkboard online platforms; Phase 2—problem review with clinical experts; Phase 3—solution pitches, formation of teams, development of prototype solutions; Phase 4—presentations and prizes awarded. The event was sponsored by ISMMS Institutes and Technology Companies. Mentors roamed throughout the event to support the teams in the technical, clinical, and business development aspects of their solutions. RESULTS/ANTICIPATED RESULTS: In total, 78 participants forming 14 teams, worked on the development of software and hardware prototypes (apps/websites, devices, wearables) to address a variety of pain and fatigue problems, culminating in final pitch presentations to a panel of judges comprised of academic experts; innovators and entrepreneurs in the technology start up space. Award recipients were: (1) PT partners, a wearable device for monitoring physical therapy post knee replacement; (2) SickleMeNot, an interactive, multimodal website/app for children designed to assess, monitor and manage pain; and (3) Biolumen, a functional biofeedback system, to treat chronic back pain. Evaluations revealed a high-degree of satisfaction with the event. Several teams continue to develop their prototypes. DISCUSSION/SIGNIFICANCE OF IMPACT: The Sinai MedMaker Challenge (1) was a compelling and productive forum to bring together students, trainees, faculty and other stakeholders to explore tech-based solutions for management, monitoring, and treatment of pain and fatigue; and (2) can be repeated annually, fostering a “Community of Practice,” and expanded to offer pre and post event opportunities to encourage iterative learning and ongoing creative output.

